# Evaluation of surveillance for surgical site infections in Thika Hospital, Kenya

**DOI:** 10.1016/j.jhin.2012.11.003

**Published:** 2013-02

**Authors:** A.M. Aiken, A.K. Wanyoro, J. Mwangi, P. Mulingwa, J. Wanjohi, J. Njoroge, F. Juma, I.K. Mugoya, J.A.G. Scott, A.J. Hall

**Affiliations:** a London School of Hygiene and Tropical Medicine, London, UK; b Kenya Medical Research Institute – Wellcome Trust Research Programme, Kilifi, Kenya; c Thika Level 5 Hospital, Thika, Kenya; d Kenyatta University, Nairobi, Kenya; e Ministry of Public Health and Sanitation, Nairobi, Kenya; f Nuffield Department of Clinical Medicine, John Radcliffe Hospital, Oxford University, Oxford, UK

**Keywords:** Epidemiology, Kenya, Sub-Saharan Africa, Surgical site infection, Surveillance

## Abstract

**Background:**

In low-income countries, surgical site infections (SSIs) are a very frequent form of hospital-acquired infection. Surveillance is an important method for controlling SSI but it is unclear how this can best be performed in low-income settings.

**Aim:**

To examine the epidemiological characteristics of various components of an SSI surveillance programme in a single Kenyan hospital.

**Methods:**

The study assessed the inter-observer consistency of the surgical wound class (SWC) and American Society of Anesthesiologists (ASA) scores using the kappa statistic. Post-discharge telephone calls were evaluated against an outpatient clinician review ‘gold standard’. The predictive value of components of the Centers for Disease Control and Prevention – National Healthcare Safety Network (CDC-NHNS) risk index was examined in patients having major obstetric or gynaecological surgery (O&G) between August 2010 and February 2011.

**Findings:**

After appropriate training, surgeons and anaesthetists were found to be consistent in their use of the SWC and ASA scores respectively. Telephone calls were found to have a sensitivity of 70% [95% confidence interval (CI): 47–87] and a specificity of 100% (95% CI: 95–100) for detection of post-discharge SSI in this setting. In 954 patients undergoing major O&G operations, the SWC score was the only parameter in the CDC-NHNS risk index model associated with the risk of SSI (odds ratio: 4.00; 95% CI: 1.21–13.2; *P* = 0.02).

**Conclusions:**

Surveillance for SSI can be conducted in a low-income hospital setting, although dedicated staff, intensive training and local modifications to surveillance methods are necessary. Surveillance for post-discharge SSI using telephone calls is imperfect but provides a practical alternative to clinic-based diagnosis. The SWC score was the only predictor of SSI risk in O&G surgery in this context.

## Introduction

A World Health Organization (WHO) systematic review in 2011 on hospital-acquired infections (HAIs) highlighted the scarcity of studies from low-income countries and from African countries in particular.^1^ From limited information, surgical site infections (SSIs) were identified as a significant problem: the risk in developing countries was ‘strikingly higher than in equivalent surgical procedures in high income countries’.

Conducting surveillance for SSIs is recognized as making an important contribution to reducing the risk of these infections. Establishing high-quality surveillance with timely feedback to surgeons can lead to reduction in risk of SSI.^2^ A systematic review of interventions for preventing SSI in sub-Saharan Africa found no examples of surveillance being conducted with the primary purpose of reducing the risk of SSI.^3^

The SENIC project (Study on the Efficacy of Nosocomial Infection Control) had demonstrated that the critical components of SSI surveillance were: (i) accurate collection and reporting of information; (ii) appropriate stratification of risk.^4^ For the first component, methods for accurate collection of information for SSI surveillance have been extensively researched in high-income countries.^5,6^ However, these methods are not applicable in hospitals in low-income settings where data extraction from inpatient records is challenging and electronic linkage to primary healthcare records is impossible. In all settings, many SSI cases occur after discharge from hospital.^6^ Incorporating these cases into surveillance systems is especially problematic in low-income settings, where surgical patients are often dispersed over a wide area.

For the second component of SSI surveillance, an appropriate system of risk stratification is needed to make comparisons of the risk of SSI between centres or over time. The Centers for Disease Control and Prevention – National Healthcare Safety Network (CDC-NHSN) risk index provides one such system. A score of 0–3 is assigned based on the sum of values derived from the American Society of Anesthesiologists physical status classification (ASA) score, the surgical wound class (SWC) and operation duration in relation to a procedure-specific length (time *T*).^7^ Although these components of the risk index are, in principle, readily transferable to low-income settings, an important question is whether accuracy of scoring in routine clinical practice in such settings is adequate to reliably predict risk of SSI.

The aim was to evaluate epidemiological characteristics of various components of SSI surveillance in a low-income hospital setting in sub-Saharan Africa. We evaluated: the inter-observer consistency of SWC and ASA scoring; the sensitivity and specificity of telephone calls for identifying SSI against a clinician review ‘gold standard’; the association of CDC-NHSN risk index components with the risk of SSI in this setting.

## Methods

Thika Level 5 Hospital is a 300-bed government hospital in the town of Thika, about 50 km north east of Nairobi, in Central Province of Kenya. At Thika Hospital there are six consultant surgeons and a rotating pool of 16–20 medical officers (junior doctors) and clinical officers (vocationally trained clinicians) who carry out a range of elective and emergency surgical procedures. There are four operating theatres and about 300 major and minor operations take place monthly. Caesarean sections are the most commonly performed procedure. Surgical instruments are reprocessed in a steam autoclave with monitoring by a change in colour of sealant tape. Prior to February 2011, antibiotic prophylaxis was normally administered to patients postoperatively, as is standard practice in many government hospitals in the region. Thika Hospital is not a university hospital, nor does it have an extensive history of research collaborations. It is a typical mid-sized Kenyan government hospital.

As a collaborative project between the Ministry of Medical Services, the Kenya Medical Research Institute and Thika Hospital, SSI surveillance was conducted at Thika Hospital for a continuous period from August 2010 to December 2011.

All patients gave written consent to participation in surveillance, which included contact by phone after discharge from hospital. This study was approved by the KEMRI National Ethical Review Committee.

Postoperative patient reviews, data and sample collection, phone calls and data entry were performed daily by a team of hospital staff members (two clinical officers and four support staff). All data for CDC-NHNS risk index criteria were recorded by hospital surgeons and anaesthetists. We diagnosed SSI in accordance with CDC-NHSN definitions as far as possible given the diagnostic facilities available.^8^ Microbiological criteria were not used for SSI diagnosis, although microbiology services at Thika Hospital were upgraded as part of the surveillance. All diagnoses of SSI were discussed with the relevant surgical team and an infectious diseases physician. Feedback of ongoing surveillance results was given in writing and discussed in a series of multidisciplinary seminars.

In our surveillance, we included all surgical operations where a surgical wound was created during the procedure and the patient stayed overnight in hospital. We therefore excluded patients having day-case surgery (including all ear/nose/throat, ophthalmic and minor gynaecological procedures) and debridement of traumatic or infected wounds. During their inpatient stay, postoperative patients were reviewed at every dressing change, normally starting on the third postoperative day and on alternate days thereafter. Patients remained in SSI surveillance for 30 days after all eligible surgical operations, including both inpatient and outpatient periods. Postoperative readmissions to Thika Hospital were actively sought daily. After discharge, telephone-based surveillance was conducted as described below. Patients were encouraged to contact the surveillance team if they received treatment for wound complications at another facility.

All information for SSI surveillance was recorded in a custom-made PHP-MySQL database. Statistical analyses were performed using Stata v12 software (Stata Corp., College Station, TX, USA).

### Consistency of SWC and ASA scores

Scoring consistency tests were conducted with the Surgery, Obstetrics and Gynaecology (O&G) and Anaesthetics departments in Thika Hospital. In each department, a series of 10 case histories was developed describing patient scenarios similar to those encountered in local practice. After revision of the relevant scoring system, all departmental members independently scored the SWC or ASA for these case histories. These results were presented to each department and the need to ensure inter-observer consistency was explained. Various different approaches to improving consistency were employed: departmental discussions, educational meetings and written guidelines and posters in departments. Among surgeons, a second round of this exercise was conducted with actual patients; in the O&G and Anaesthetics departments, a second round of this exercise used different paper-based case histories.

As the scoring depended on making a clinical judgement, no answer was considered ‘correct’: the kappa statistic (κ) was employed to assess inter-observer consistency. A weighted κ was used to account for the ordered nature of the scoring categories.

### Sensitivity and specificity of telephone-based surveillance

At discharge, patients (or their guardians) were asked to give a mobile phone number where they could be contacted during the next 30 days. More than 90% of patients provided this. Patients were contacted twice by mobile phone on approximately the 14th and 28th postoperative days to inquire about wound complications since discharge. Surveillance staff asked a standard series of questions regarding current condition of the wound, and distinguished between presence of (normal) postoperative mild pain, itching and serous ooze and (abnormal) severe pain, wound breakdown and purulent discharge. Patients could also contact the SSI surveillance team by phone. Patients reporting current symptoms consistent with wound infection were asked to re-attend Thika Hospital for free outpatient review.

Using clinician review in the outpatient clinic as the diagnostic gold standard, paired observations of telephone interview and direct clinician review were analysed when these were performed within 48 h of each other to determine the sensitivity and specificity of telephone calls as a diagnostic test. It was assumed that ‘wound infection status’ could not change within a 48 h period.

### Performance of CDC-NHSN risk index

To analyse the predictive performance of the CDC-NHNS risk index, all forms of SSI were re-categorized (including those occurring after discharge) into a single outcome variable. Exposure variable data were extracted from medical notes at the time of the operation. Logistic regression was used to analyse risk index components in a group consisting of caesarean sections and major gynaecological operations conducted between August 2010 and February 2011. Time *T* represented 1 h for all of these procedures. We did not include other procedures in this modelling as the risk index is only intended to be applied to groups of similar procedures – there were insufficient numbers of any other operative group for adequate evaluation.

## Results

Following extensive development, training and piloting, SSI surveillance was conducted at Thika Hospital for a 16-month continuous period from August 2010 to December 2011). All consecutive adult and paediatric patients undergoing eligible operative procedures were enrolled.

### Consistency of SWC and ASA scores

Consistency studies were conducted for the SWC and ASA score as shown in Table I. Junior staff rotated between departments every three months, so this exercise could not be repeated in the surgical departments at intervals longer than this. The second exercise was performed in the Anaesthetics department after a 13-month interval.

Whereas consistency of scoring between clinicians initially ranged from fair (within the O&G department) to excellent (within the Surgery department), after a period of routine use of these scoring systems, the consistency between clinicians improved in all departments. The high degree of consistency achieved in the Surgery department in a ‘paper-based’ exercise (κ = 0.81) was replicated when the same approach was applied with actual patients (κ = 0.83, based on 55 paired observations). Subsequently, continuous training of incoming staff was used to maintain these high levels of consistency.

### Validity of telephone-based surveillance

There were a total of 89 pairs of outpatient clinician reviews and telephone interviews within 48 h of each other. For 23 patients diagnosed in outpatients by a clinician as having SSI, 16 of these had been judged to have SSI in their telephone interview. For 66 patients seen in outpatients by a clinician and considered not to have SSI, none of these had been considered to have SSI in their telephone interview. On the basis of these results, the sensitivity of telephone calls was 69.6% [95% confidence interval (CI): 47.1–86.8%] and the specificity was 100% (95% CI: 95–100%).

### Description of surveillance operations and performance of CDC-NHSN risk index

Between August 2010 and February 2011, a total of 1172 operations conducted at Thika Hospital were followed up in SSI surveillance. The characteristics of these operations are given in Table II. As caesarean sections predominated among these procedures (75% of operations), surveillance mainly included women of child-bearing age. A wide variety of other operations were also included in surveillance including laparotomies, hysterectomies, hernia repairs, appendicectomies and amputations. There was a high risk of SSI in orthopaedics and neurosurgery (14%), reflecting the high incidence of extensively contaminated road-accident trauma in this hospital. Surgery performed by clinical officers (vocationally trained clinicians) did appear to have elevated risk of SSI (15%) although this is based on a very small number of procedures. Antibiotic prophylaxis was normally delivered as a postoperative regime, as is widely used in hospitals in Kenya. We have not calculated an overall risk of SSI in the surveillance cohort – this is strongly dependent on the mix of procedures, which is outside of institutional control.

The performance of the CDC-NHSN risk index in predicting the outcome of SSI (of any type, including post-discharge infections) in caesarean sections and major gynaecological operations is given in Table III. For individual components of the risk index, only the parameter derived from the SWC (odds ratio: 3.93; 95% CI: 1.25–12.4; *P* = 0.02) was significantly associated with the risk of SSI – this remained the case in a multivariate model. Parameters relating to the ASA score and the duration of the operation were not associated with the risk of SSI in these operations.

## Discussion

There are very few previous reports that have studied SSI surveillance methods in sub-Saharan Africa, and we are unaware of any that have examined either the consistency of scoring of risk index components or the characteristics of telephone calls as a surveillance method for SSI.^9,10^ This study represents the largest SSI surveillance cohort reported in sub-Saharan Africa.

After appropriate training and with ongoing feedback, surgeons and anaesthetists in Thika Hospital were consistent in categorization of SWC and ASA scores in paper-based evaluations. This consistency improved over time and was similar when applied to actual patients. On this basis, the scoring of these indices by clinicians in this setting can achieve an adequate consistency to make them suitable for use as SSI risk stratification variables.

The sensitivity and specificity of telephone calls as a post-discharge follow-up tool for SSI surveillance were 70% and 100% respectively. Although this was based on a relatively small number of paired observations, the analysis was restricted to those observations within 48 h of each other to maximize accuracy. Specificity was high as there were no false-positive telephone-based diagnoses (type 1 errors) among our paired observations. The moderate sensitivity obtained means that some genuine SSI cases may have been missed (type 2 errors) in surveillance. This combination of high specificity and moderate sensitivity are satisfactory characteristics for a ‘stand-alone’ test where the outcome is rare. During the pilot phase of surveillance, we had found that in Thika Hospital the actual attendance at postoperative outpatient appointments was extremely poor (<25%). Researchers in Tanzania had also found poor attendance (54%) at postoperative follow-up appointments.^9^ Telephone calls have also been used for post-discharge SSI detection in a cluster-randomized trial of a surgical hand-washing intervention in Kenya, although that study did not examine the sensitivity or specificity of the method.^11^ Telephone calls appear to be a reasonable method of detecting post-discharge SSI in this setting, although this should be further evaluated as a diagnostic tool.^12^ This method may be population-specific as Kenya has very high penetration of mobile phone services.

Surgical wound class was predictive of risk of SSI in the univariate and multivariate models in major O&G operations. This is consistent with studies from Tanzania and Ethiopia and with findings from elsewhere in the world, where SWC remains a cornerstone of SSI risk stratification.^1,8,10,13^

Few studies in Africa have examined the use of the ASA score as a predictor of SSI risk. The ASA score approximates ‘global’ patient health at the time of the operation, and is a reliable predictor of the risk of SSI in high-income settings.^14^ However, it provided no useful predictive information in this setting when applied to major O&G operations. This may be because these procedures were mainly performed on healthy young women among whom higher scores were rarely assigned, which may limit the utility of the ASA score as a discriminator of risk. In other African studies, the ASA score was strongly predictive of risk in a mixture of obstetric and general surgical procedures in Tanzania but only weakly predictive of risk in a study of paediatric surgery in Nigeria.^10,15^

The duration of an operation gives an indication of the complexity of performing that specific procedure. When this is compared to a standard length, this can predict the risk of subsequent complications. Operation duration was predictive of SSI risk in several studies in low-income settings, though using ‘local’ values for standard operation length was found to improve the predictive power.^9,10,16,17^ The duration of surgery in this study was not associated with the risk of SSI in major O&G operations.

There are limitations to our study. Both the evaluation of the consistency of SWC and ASA scores and the sensitivity and specificity of telephone calls are based on relatively small numbers of observations. However, these are the most detailed examinations of these topics in the region to date. The evaluation of the CDC-NHNS risk index in O&G surgery is the largest such group ever reported in sub-Saharan Africa.

Six full-time staff with salaries equivalent to entry-level nurses were required to conduct this surveillance. About 250 procedures were followed up each month. The high cost of supporting these staff meant that surveillance could not be sustained beyond the duration of this collaborative project. Commercially available software for SSI surveillance might have been a cheaper alternative to our bespoke, locally programmed database.

The value of surveillance is providing surgeons with comparative data about their specific performance. Combining many different types of procedure with different intrinsic risks of infection is therefore of no real value. In the smaller throughput types of surgery, it would be necessary to collect surveillance data for much longer periods or across many sites in order to adequately evaluate the risks of SSI.

In conclusion, surveillance is an important method of measuring and controlling SSIs, which are recognized to be a significant form of HAI in sub-Saharan Africa. In a low-income hospital setting it was found that surgeons and anaesthetists can assign consistent scores for the SWC and ASA; ongoing training and feedback are required. Telephone calls appear to be a reasonable method of detecting post-discharge SSI cases, although further evaluation is needed. Of the components of the CDC-NHNS risk index, only the SWC provided useful predictive information on the risk of SSI in our hospital, where caesarean sections represent the bulk of operations.

## Figures and Tables

**Table I:** Results of scoring consistency projects

Department	Test	No. of staff	First exercise		Second exercise^a^	
			Date	Average weighted κ score	Date	Average weighted κ score
Anaesthetics	ASA	5^b^	Jun 2010	0.68	Sep 2011	0.89
O&G	SWC	6	Jun 2010	0.48	Sep 2010	0.72
Surgery	SWC	7	Jun 2010	0.81	Aug–Oct 2010	0.83

ASA, American Society of Anesthesiologists; SWC, surgical wound class. There are no *P*-values associated with κ as it does not test a particular hypothesis.

a All exercises compared paper-based scenarios except the second exercise in surgery which examined actual patients.

b Only four of the five participants undertook the second exercise.

**Table II:** Risk of surgical site infection (SSI) in operations at Thika Hospital, Kenya: August 2010–February 2011

Variable	Total no.	SSI events (% of all)
Patient age (years)		
≤14	32	0 (0)
15–39	1015	76 (7.5)
40–65	110	16 (14.6)
≥65	15	1 (6.7)
Patient sex		
Female	1060	82 (7.7)
Male	112	11 (9.8)
Type of surgery^a^		
Caesarean section	882	64 (7.3)
General surgery	144	12 (8.3)
Orthopaedic/neurosurgery	36	5 (13.9)
Gynaecological	110	12 (10.9)
Surgeon grade		
Consultant	198	17 (8.6)
Medical officer	685	51 (7.5)
Medical officer intern	266	21 (7.9)
Registered clinical officer	20	3 (15.0)
Preoperative antibiotic prophylaxis^b^		
Given	18	2 (11.1)
Not given	1154	91 (7.9)
Postoperative antibiotic prophylaxis/treatment^c^		
Given	1169	91 (7.8)
Not given	3	1 (33.3)
Total operations followed up	1172	93 (7.9)

a Includes caesarean section with tubal ligation (*N* = 36) and with hysterectomy (4); general surgery includes laparotomy (61), hernia repair (19), appendicectomy (10); orthopaedic/neurosurgery includes open reduction internal fixations (9), limb amputations (7) and plating procedures (5); gynaecological surgery includes hysterectomy (43), salpingectomy (11), cystectomy (8).

b Antibiotics administered in the 60 min before the start of surgery.

c Any antibiotic prescription written up to start/continue immediately after surgery.

**Table III:** CDC-NHSN risk index in Thika Hospital, Kenya in major Obstetrics and Gynaecology surgery^a^ (*N* = 954)

Variable	Total no.	SSI (any)	Individual component		Full risk index	
			OR (95% CI)	*P*-value	OR (95% CI)	*P*-value
Surgical wound class						
Clean	103	9				
Clean-contaminated	834	59				
Contaminated	13	2				
Dirty-infected	4	2				
CDC-NHSN categorization						
Clean or clean-contaminated	937	68	1.0		–	
Contaminated or dirty-infected	17	4	3.93 (1.25–12.4)	0.02	4.00 (1.21–13.2)	0.02
ASA score						
ASA 1	629	44				
ASA 2	294	25				
ASA 3	27	3				
ASA 4–5	4	0				
CDC-NHSN categorization						
ASA 1–2	923	69	1.0		–	
ASA 3–5	31	3	1.32 (0.39–4.47)	0.65	0.88 (0.24–3.22)	0.85
Operation duration (min)						
<30	162	9				
30–60	616	46				
60–120	171	16				
≥120	5	1				
CDC-NHSN categorization						
<60 min	778	55	1.0		–	
≥60 min	176	17	1.41 (0.79–2.49)	0.24	1.25 (0.77–2.04)	0.37
Total operations	954	72				

CDC-NHSN, Centers for Disease Control and Prevention – National Healthcare Safety Network; SSI, surgical site infection; OR, odds ratio; CI, confidence interval; ASA, American Society of Anesthesiologists.

a Includes all types of caesarean section, hysterectomy, salpingectomy, cystectomy and oophorectomy.
